# Choosing Mastectomy vs. Lumpectomy-With-Radiation: Experiences of Breast Cancer Survivors

**DOI:** 10.7759/cureus.18433

**Published:** 2021-10-02

**Authors:** Claudia Admoun, Harvey Mayrovitz

**Affiliations:** 1 Surgical Oncology, Nova Southeastern University Dr. Kiran C. Patel College of Osteopathic Medicine, Fort Lauderdale, USA; 2 Medical Education, Nova Southeastern University Dr. Kiran C. Patel College of Allopathic Medicine, Davie, USA

**Keywords:** breast reconstruction revision, chronic pain, radiation, complications, surgery, lumpectomy, mastectomy, breast cancer

## Abstract

Background

Annually about 280,000 women are diagnosed with breast cancer. Treatment options depend on age, comorbidities, tumor stage, grade, size, and other factors. Often, patients must decide between two surgical treatment options: mastectomy or lumpectomy-with-radiation herein simply called a lumpectomy. Since both offer similar survival outcomes, the choice ultimately is the patient’s. However, most rely on inputs from doctors, family, friends, personal research, and other actions. We believe decision-making processes for future patients will be aided if experiences of prior breast cancer survivors are known. This study’s aim is to provide such information.

Methods

Feedback from prior breast cancer survivors was obtained using a 19-question survey distributed online to multiple breast cancer support groups. It focused on issues relevant to choosing between the two surgical options including, post-surgical complications, breast reconstruction, chronic pain, cosmetics, and surgery-choice satisfaction.

Results

Respondents (N=1606) had a median age of 49 years (range 26 to 88 years) and had a median body mass index (BMI) of 26.6 Kg/m^2^. There were 978 mastectomy patients (60.9%) and 628 lumpectomy patients (39.1%). With regard to post-surgical reconstruction, 64.2% of mastectomy respondents and 13.5% of lumpectomy respondents decided to undergo breast cancer reconstruction following breast cancer surgery. Almost all (99.8%) of lumpectomy respondents had radiation side-effects; with skin irritation and thickening and chest wall tenderness being the most common. Among mastectomy patients, 94.3% had one or more complications; loss-or-changes in nipple or breast sensation, uneven breasts, chest wall tenderness, and breast swelling were the most common complications. Post-surgical pain lasting six months or more was experienced by a smaller percentage of mastectomy vs. lumpectomy patients (64.1% vs. 78%, p <0.00001). Mastectomy patients were also less likely to have pain that was persistent and present up to the time of the survey (35.4% vs. 46.0%, p=0.0002). With respect to cosmetic outcomes, mastectomy patients vs. lumpectomy patients were less likely to be either satisfied or very satisfied (52.2% vs. 62.7%, p=0.00004). Overall satisfaction of surgical treatment was 70.9% for mastectomy patients and 68.6% for lumpectomy patients.

Conclusion

Based on the experiences of these breast cancer survivors, mastectomy is associated with less chronic pain frequency and lower incidence of post-surgical side effects compared to lumpectomy. However, mastectomy is associated with lower cosmetic satisfaction. Breast cancer survivors that underwent a lumpectomy, reported being more satisfied with cosmetic outcomes but almost all reported radiation side-effects with skin thickening listed as the most common. Lumpectomy was also associated with higher chronic pain frequency compared to mastectomy.

The overall surgical treatment satisfaction reported by mastectomy and lumpectomy respondents was similar. The composite findings will provide information that will aid future breast cancer patients in making a decision between having a mastectomy or a lumpectomy.

## Introduction

Breast cancer is the second most common cancer for women in the United States following skin cancer [1]. There is a 12.3% average risk that a woman in the U.S. will get breast cancer in her lifetime. This translates to one in eight women would be diagnosed with breast cancer. Due to advancing preventative measures and improved personalized treatment options, breast cancer survival rates have been steadily increasing [2]. The five-year survival rate is 100% for stage 0 and I breast cancer, 93% for stage II, 72% for stage III, and 22% for stage IV [3].

Surgery is the treatment of choice for most early-stage breast cancer cases [4]. It is used as a curative treatment option that is done along with chemotherapy, radiation, or hormonal treatment options when indicated. The National Surgical Adjuvant Breast and Bowel Project (NSABP) was a landmark study in the surgical treatment options of breast cancer [5]. This study along with other consequential studies [6-8] have shown that there is no difference between mastectomy vs. lumpectomy alone vs. lumpectomy with radiation when it comes to survival rates [5]. However, the studies showed that the recurrence rates were increased significantly in patients who underwent lumpectomy without radiation. The overall breast cancer recurrence rate of patients who underwent lumpectomy without radiation was 39.2% compared to patients that underwent lumpectomy with radiation who had a recurrence rate of 14.3% [5]. Thus, when comparing mastectomy and the breast-conserving therapy (BCT) of lumpectomy, studies have shown that, in most circumstances, there is no difference in both overall survival and breast cancer recurrence rate [4].

Recent studies, however, suggest that breast-conserving surgery is associated with increased survival rates for early-stage breast cancer compared to mastectomy [9, 10]. When examining these findings, it is important to consider the impact of factors such as socioeconomic status, existing comorbidities, rates of adjuvant chemotherapy, and stage at presentation on one's survival rates. None of these variables, which present a significant effect on survival rates, were considered in these studies. This suggests that their findings of lower survival rates in patients who underwent a mastectomy compared to those who opted for a breast-conserving surgery can be due to the existence of other variables that lead to worse survival outcomes [9, 10]. The reason for the difference in survival rates that were found in these studies has not yet been determined.

Upon diagnosis, breast cancer patients are presented with individualized treatment options that are tailored specifically for them. The treatment is based on many factors including the patient’s age, overall health, and the patient’s preferred route of medical care along with the breast cancer’s type, size, stage, and grade. Surgical options are also based on the tissue’s resection volume compared to breast volume and the location of the tumor [11]. 

Many breast cancer patients are presented with two surgical treatment options which are mastectomy and a breast-conserving surgery option in the form of lumpectomy. The choice between these two surgical treatment options is individualized and based on medical recommendations along with the patient’s personal preference [12].

The purpose of the present study was to obtain comparative information to help guide breast cancer patients who will be deciding between having a mastectomy or lumpectomy. This study was designed to investigate the factors that prospectively and then retrospectively influenced patients’ decision making and satisfaction with their decisions considering breast cancer recurrence, chronic pain, cosmetic results, surgical side effects, radiation side effects, breast reconstruction success rate, and overall patient satisfaction.

## Materials and methods

Participants

The data from this study was obtained via survey from breast cancer survivors who had undergone either a mastectomy or lumpectomy-with-radiation. An anonymous survey was created and subsequently approved by Nova Southeastern University Institutional Review Board (IRB No.: 2021-156-NSU). The survey included a participation letter naming the individuals conducting the study and explaining the purpose of this research project. It was posted on the following breast cancer support group platforms: Facebook breast cancer groups, Breastcancer.org, and Cancer Survivors Network. It was made evident that the survey is to be completed once in case there was an overlap of members in these support groups. The members of these support groups include female breast cancer survivors, current female breast cancer patients, women with a family history of breast cancer concerned about getting diagnosed, and family members supporting those affected. There were 1606 respondents to the survey with 978 reporting they had had a mastectomy and 628 reporting that they had a lumpectomy. 

Survey questions

The survey included 19 questions (Table 1) with the goal of obtaining information regarding the participant's breast cancer diagnosis along with their most recent breast surgery type and date. The questions were designed to investigate if patients made their surgical option decision with sufficient knowledge of the aftermath of these two surgeries. The questions also investigated the source of influence in making their decision and if the expected cost of the procedure played a factor in determining the patient’s decision. The survey then proceeded to ask questions regarding the aftereffects of the surgical procedure including breast cancer recurrence, side effects of possible radiation and reconstruction, the incidence of chronic pain following the procedure, cosmetic effects in terms of post-surgery scars, and overall satisfaction of their choice of breast cancer surgery. The data were collected through Google forms anonymously and exported to Microsoft Excel.

**Table 1:** Survey questions and answer choices presented to breast cancer survivors

Survey Questions	Answer Options
1) In what year did you have your most recent breast cancer surgery done?	Pull down selection
2) Why did you receive breast surgery?	Diagnosed with unilateral breast cancer, bilateral breast cancer, or was the treatment done prophylactically
3) What was the breast cancer stage?	DCIS, LCIS, Stage 1, stage 2, stage 3, stage 4, treatment was done prophylactically
4) What was your most recent breast cancer surgical treatment?	Lumpectomy with whole-breast radiation, lumpectomy with partial breast radiation, single mastectomy, double mastectomy, nipple-sparing single mastectomy, nipple-sparing double mastectomy, mastectomy following prior lumpectomy with radiation
5) What was your approximate age when you had your surgical operation?	Pull down selection
6) What was your approximate weight when you had your surgical operation?	Pull down selection
7) What was your approximate height when you had your surgical operation?	Pull down selection
8) When did you make your decision between having a mastectomy or a lumpectomy with radiation?	No choice given - a mastectomy was required, no choice given - a lumpectomy with radiation was required, during the consultation visit after being presented with the two options, following the consultation visit after thorough thought or research, do not remember
9) What factor(s) importantly influenced your decision? (Can pick multiple)	Doctor, family, friend, online research, support group, other
10) Was your decision influenced by the expected cost of a procedure?	Yes or No
11) Did you have breast cancer reoccurrence following your most recent surgical breast operation?	Yes or No
12) If you underwent radiation, did you have any of the following radiation side effects? (Can pick multiple)	Did not undergo radiation, no side effects, loss or change in nipple or breast sensation, breast swelling, uneven breasts, skin irritation, skin thickening, rib fracture, chest wall tenderness, non-healing wound ulcer
13) How many axillary lymph nodes were removed?	0, 1-3, 4-6, 7-9, 10+, Do not remember
14) Did you undergo reconstruction following your surgical operation?	Yes or No
15) If you underwent breast reconstruction, did you have any of the following complications? (Can pick multiple)	Did not undergo reconstruction, no complications, loss or changes in nipple or breast sensation, breast swelling, uneven breasts, infection, breast tissue death (necrosis), problems with breast implant(s), hematoma formation, non-healing ulcer
16) Starting from six months after your surgery, did you experience pain at the breast, chest, armpit or arm?	No; yes but no longer present; yes, it is still painful
17) Referring to the above question (16), what was the severity of the pain?	No pain, mild pain, moderate pain, severe pain, very severe pain, worst possible pain
18) Cosmetically, are you satisfied with your post-surgery scars?	Very satisfied, Satisfied, OK, Dissatisfied, Very dissatisfied
19) Based on the surgical treatment option you chose, are you satisfied with your decision or would you have done it differently?	Very satisfied, Satisfied, OK, Dissatisfied, Very dissatisfied, No choice given
Optional comment section	

Analysis

The data set was stratified based on surgery type and the presence or absence of a choice of the prospective surgery based on “no choice was given” answers. All statistical analyses were done using SPSS version 16 (Chicago, IL, SPSS Inc.). Statistical differences between mastectomy and lumpectomy respondents with respect to age, age at surgery, years from surgery, and body mass index (BMI) were tested using independent t-tests. Chi-square tests were used to determine if there was a statistically significant difference in all other responses including tests to determine differences in which surgical choice was made; mastectomy vs. lumpectomy. Parameters assessed included post-surgical complications, breast reconstruction, chronic pain, the incidence of breast cancer reoccurrence between breast cancer surgery and time of the survey, cosmetic satisfaction rate, and overall satisfaction with surgical treatment. Statistical differences were taken as significant if p-values were less than 0.05. 

## Results

Respondent information

There were 1606 respondents whose demographics are summarized in Table 2. Breast cancer survivors who reported undergoing a mastectomy were on average about three years younger at the time of surgery and at the time of survey compared to those who underwent a lumpectomy. There was a statistically significant difference between the BMI of the two groups where the average BMI in mastectomy respondents was 27.1 and the average BMI in lumpectomy patients was 28.2. The reason for surgery was predominantly the presence of stage I and II unilateral breast cancer among respondents. Among ductal carcinoma in situ (DCIS) patients, a significant number of respondents chose a mastectomy instead of a lumpectomy.

**Table 2:** Respondent information Entries describing patient demographics are listed as means ± SD. Cancer stages and reasons for surgery data describe the number and (percentage) of responses. DCIS: Ductal carcinoma in situ; LCIS: Lobular carcinoma in situ

Number of Respondents	1606		
Type of surgery	Mastectomy	Lumpectomy	P-value
Total respondents	978	628	-
Age at surgery (Years)	48.1 ± 9.3	51.3 ± 9.6	<0.0001
Age at survey (Years)	51.3 ± 9.6	51.4 ± 9.8	<0.0001
Years from surgery	3.3 ± 4.0	3.4 ± 4.0	0.752
BMI (Kg/m^2^)	27.1 ± 6.4	28.2 ± 7.1	<0.001
Cancer Stages			
Stage 1	293 (51.3)	278 (48.7)	0.763
Stage 2	314 (60.6)	204 (39.4)	0.743
Stage 3	193 (81.4)	44 (18.6)	0.001
Stage 4	18 (94.7)	1 (5.3)	0.001
Stage 0 (DCIS)	140 (59.6)	95 (40.4)	0.665
Stage 0 (LCIS)	6 (50.0)	6 (50.5)	0.555
Prophylactic	14 (100.0)	0 (0.0)	-
Reason for surgery			
Unilateral breast cancer	843 (60.3)	555 (39.7)	0.223
Bilateral breast cancer	117 (64.6)	64 (35.4)	0.093
Prophylactic	18 (66.7)	9 (33.3)	0.691

Timing of surgical choice

There were 1064 respondents who had an unrestricted choice between a mastectomy or a lumpectomy, 364 had no choice but to undergo a mastectomy, and 178 had no choice but to undergo a lumpectomy (Table 3). Mastectomy was chosen by 614 and lumpectomy was chosen by 450. A small number of respondents could not recall if given a choice or when the choice was made. Respondents choosing a mastectomy made this choice more frequently after the physician consult rather than during the consult. This pattern was reversed for respondents who chose to have a lumpectomy. The differences between the timing of the surgical choice among the respondents who had an unrestricted choice were statistically significant (p=0.0022). 

**Table 3:** When respondents made their surgical choice Entries are the number of responses. Unrestricted choice refers to patients who had no restriction for a surgical or medical reason.

	Mastectomy	Lumpectomy	P-value
Total respondents	978	628	-
Timing of choice			
During consult	264	237	0.002
After consult	337	206	0.002
No choice	364	178	<0.001
Unknown	13	7	0.709
Surgical choice			
Unrestricted choice	614	450	0.100
Restricted choice	364	178	0.100

Factors influencing surgical choice

**Figure 1:**
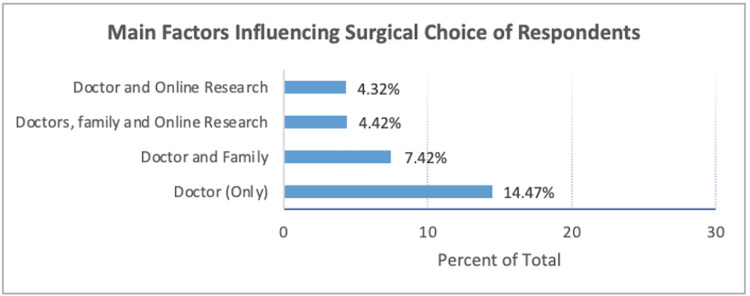
Factors influencing surgical choice Respondents constitute those with unrestricted choice only. Entries are percentages indicating the top four selected influencing factors.

External factors that influenced surgical choice (Figure 1) were reportedly the consulting doctor along with family and online research. The choices presented to participants were doctor, family, friend, online research, support group, and other. The data presented excludes 12.41% of participants who chose “other” as a response.

Surgery characteristics

Of the 1606 respondents, 978 reported having had a mastectomy, and 628 reported undergoing a lumpectomy (Table 4). Even though 86.2% of mastectomy breast cancer survivors reported unilateral breast cancer as the reason for surgery, 66.3% of mastectomy respondents underwent a double mastectomy. Among lumpectomy respondents, 74.7% underwent whole breast radiation instead of partial breast radiation. Intra-operative lymph node removal was reported by 87.9% of all responders with the removal of one to three nodes being the most common. Among overall respondents, 46.6% had removal of one to three nodes, 16.1% had removal of four to six nodes, 6% had removal of seven to nine nodes and 19.2% had removal of 10 or more lymph nodes. An overall 9.4% of respondents reported no lymph node removal and 2.7% do not remember the number, if any, of lymph nodes removed. 

**Table 4:** Surgery characteristics Entries for mastectomies and lumpectomies present the number and (percentage) of overall respondents. 'Nodes removed' represent the number of lymph node(s) that were removed during surgery.

MASTECTOMIES	
Single	263 (16.4)
Double	537 (33.4)
Nipple-sparring single	23 (1.4)
Nipple-sparring double	111 (6.9)
After prior lumpectomy	44 (2.7)
LUMPECTOMIES	
With whole-breast radiation	469 (29.2)
With partial-breast radiation	159 (9.9)
NODES REMOVED	
0	151 (9.4)
1 - 3	748 (46.6)
4 - 6	258 (16.1)
7 - 9	96 (6.0)
10+	309 (19.2)
Unknown	44 (2.7)

Complications following surgery

**Figure 2:**
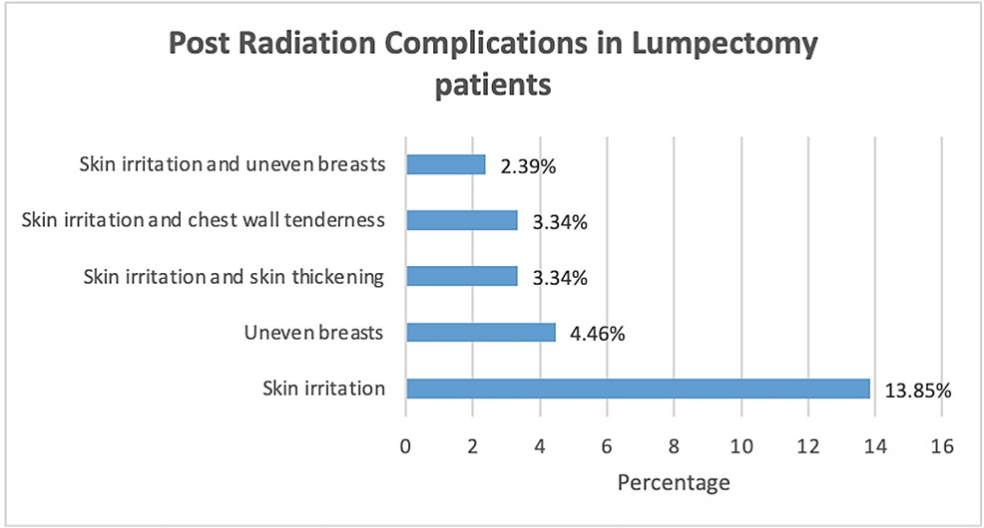
Radiation side effects in lumpectomy patients The graph represents the top radiation side effects reported by lumpectomy respondents

Among lumpectomy patients, 98.0% reported experiencing side effects following radiation (Figure 2), with skin irritation, chest wall tenderness, and skin thickening being the most common.

**Figure 3:**
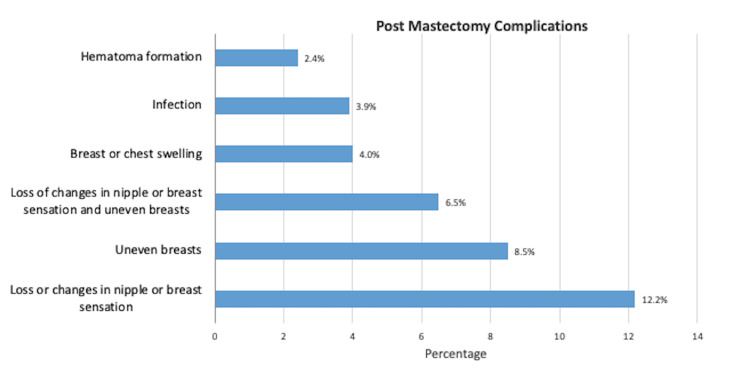
Post mastectomy complications The graph represents the top six post-surgical complications reported by mastectomy respondents.

Among mastectomy patients (Figure 3), an overall 92.9%, in which 47.5% had mastectomy alone and 52.2% had a mastectomy with reconstruction, reported having at least one complication following surgery. Loss or changes in nipple or breast sensation, uneven breasts, and breast swelling were the most common.

Post-surgical outcomes

Breast reconstruction was reported by 64.2% of mastectomy and 13.5% of lumpectomy-with-radiation patients (Table 5). Further analysis indicated that 52.2% mastectomy and 62.7% lumpectomy patients were satisfied or very satisfied with their post-surgical scar outcome cosmetically. Patients overall satisfaction of their perspective choice of surgical treatment when considering all aspects of their experience showed that 70.9% mastectomy and 68.6% lumpectomy patients were satisfied or very satisfied.

**Table 5:** Post-surgical outcomes Table entries are numbers of respondents (in percentage). Chronic pain findings were limited to (N=998) respondents. Satisfaction rates represent those satisfied or very satisfied with the outcome. Cosmetic satisfaction was asked in the context of post-surgical scar satisfaction.

	Mastectomies			Lumpectomies		
Type	No reconstruction	With reconstruction	P-value	No reconstruction	With reconstruction	P-value
Total	350 (35.8)	628 (64.2)	-	543 (86.5)	85 (13.5)	-
Complications	196 (56.0)	434 (69.1)	< 0.001	533 (98.2)	83 (97.6)	0.749
Chronic pain	232 (66.3)	401 (63.8)	0.445	429 (79.0)	66 (77.6)	0.776
	Overall Mastectomies			Overall Lumpectomies		
Cosmetic satisfaction	511 (52.2)			394 (62.7)		0.030
Overall satisfaction	693 (70.9)			431 (68.6)		0.689

Chronic pain following surgery

Around 70.2% of participants, including 64.7% of mastectomy and 78.8% of lumpectomy patients, experienced post-surgical chronic pain as note above in Table 5. Post-surgical chronic pain was defined to respondents as pain at the breast, chest, armpit, or arm that was experienced six months after surgery. An overall 35.4% of mastectomy and 46.0% of lumpectomy patients experienced pain that was persistent and present up to the time of the survey. Chronic pain severity had a range from mild to worst possible pain (Table 6). Pain severity was reported as 46.5% mild, 44.9% moderate, 7.9% severe or very severe, and 0.2% experienced worst possible pain with similar findings among mastectomy and lumpectomy respondents.

**Table 6:** Chronic pain severity Entries are the number (percentage) of responses. Respondents included here are those who reported chronic pain at the breast, chest, armpit, or arm, six months after surgery.

Pain Severity	Mastectomy	Lumpectomy	P-value
None	349 (35.8)	135 (21.5)	< 0.001
Mild	294 (30.0)	246 (39.2)	< 0.001
Moderate	284 (29.0)	215 (34.2)	< 0.028
Severe	40 (4.1)	26 (4.1)	< 0.961
Very severe	10 (1.0)	5 (0.80)	< 0.645
Worst possible	1 (0.10)	1 (0.20)	-

## Discussion

The goal of our study is to provide pertinent information for breast cancer patients when deciding between mastectomy vs. lumpectomy. Information that is based on the experiences of breast cancer survivors. Responses were obtained via a 19-question survey that was distributed online to multiple breast cancer support groups. Our findings indicate that there were significant differences between mastectomy and lumpectomy with regard to cosmetic outcomes and pain frequency. Lumpectomy patients were significantly more satisfied with their cosmetic outcomes following surgery in terms of scar appearance but reported post-radiation side effects of skin thickening. Mastectomy patients had significantly less pain frequency in terms of six months or more threshold of post-surgical chronic pain outcomes. Obtained data from this study such as those showing statistically significant differences between post-surgery cosmetic outcomes and pain frequency when comparing mastectomy with lumpectomy can aid future breast cancer patients with their decision making.

Choosing between mastectomy vs. lumpectomy depends on several factors. Our research indicated that among the 1064 respondents who had an unrestricted choice between a mastectomy and lumpectomy, 56.9% decided to undergo a mastectomy while 43.1% decided to undergo a lumpectomy. The decision was mostly influenced by the consulting physician and the timing of the decision was evenly distributed between making their choice during the consultation visit and after the consultation visit. This is in line with several study findings suggesting consulting physicians have a very important input in the choice of surgery type [4]. Research suggests that the reasons to choose mastectomy include having multiple sites of cancer in different locations of the breast, having a larger cancerous tumor relative to the size of the breast, not being a candidate for radiation due to previous breast radiation or pregnancy, wanting to avoid radiation, having a genetic mutation that increases the risk of cancer, or wanting to avoid annual mammograms and MRIs on the operated breast(s) [13]. Our study is in line with study findings suggesting that more people are opting to have double mastectomies when choosing mastectomy as their surgical choice [14]. In most cases, however, having cancer in one breast does not significantly increase the risk of having cancer in the other breast [14]. The average risk of breast cancer survivors developing breast cancer on the opposite breast is estimated to be 0.5% annually with the risk decreasing significantly if the patient had undergone chemotherapy or hormone therapy as part of their treatment plan [14]. Factors such as having lobular breast cancer, younger age at diagnosis, estrogen receptor-negative tumor, family history of breast cancer, and BReast CAncer (BRCA) gene mutation increase the likelihood of developing breast cancer on the contralateral breast [14-16]. Conversely, reasons to choose lumpectomy include having a small tumor, not having previously undergone radiation on the operated breast, conserving the breast, conserving more breast sensation, and accepting the risk of needing more surgery if there are positive margins on the tumor or if breast cancer reoccurs on the operated breast [4].

Pain has been reported as the most frequent and debilitating symptom following breast cancer surgery [17]. Chronic pain impacts patients emotionally and affects their physical function and quality of life [18]. Lumpectomy does not show a reduction in post-surgical chronic pain compared to mastectomy, and several studies have shown that there might be more cases of persistent pain post lumpectomy compared to mastectomy [19, 20]. Our research supported the previous findings of higher rates of chronic pain post lumpectomy. Our data indicate a significantly higher percentage of lumpectomy breast cancer survivors experiencing post-operative persistent chronic pain compared to those who underwent a mastectomy. According to prior studies, the reason for the persistent chronic pain has been suggested to be caused by either post-surgical changes to neuroendocrine or inflammatory systems or due to central nervous system sensitizing effects [21]. There are also findings indicating that persistent chronic pain post breast cancer surgery causes alterations in pain-modulatory processes in the brain [22].

Radiation for breast cancer, which usually is a six-week post-surgical treatment, is almost always needed after lumpectomy [4]. However, having a mastectomy makes a follow-up radiation less likely and is only recommended for more advanced or pathologically high-risk breast cancer [23]. The need for radiation following mastectomy may not be known prior to surgery since it depends on cancer pathology and nodal involvement which are obtained during surgery [23]. Radiation side effects can be classified into early, intermediate, and late complications [24]. Reports suggest that skin thickening, breast edema, fat necrosis, pneumonia, and pleura effusion are considered early post-radiation side effects experienced weeks to months post-surgery. Skin retraction with breast fibrosis, glandular atrophy, lactation difficulty, overlying bone fracture, pulmonary fibrosis, and pericardial disease are considered intermediate post-radiation side effects. Late side effects which can occur more than 10 years post-radiation include cardiomyopathy and radiation-induced malignancy [24]. Our research was in line with current studies and indicated that the most common radiation side effect presentations among the breast cancer survivors who had a lumpectomy with a median of 2.0 years following surgery were skin irritation, uneven breasts, skin irritation with chest wall tenderness, or skin thickening, and skin irritation along with uneven breasts.

Mastectomies include single or double total mastectomy and single or double nipple-sparing mastectomy [13]. Patients have the choice to forgo reconstruction, undergo a delayed reconstruction, or undergo immediate reconstruction at the time of mastectomy. Mastectomy is well tolerated by patients with low morbidity and mortality [13]. Post-mastectomy complications that have been reported include seroma, hematoma formation, wound infection, skin flap necrosis, and lymphedema [13]. Our study suggests similar findings with the main presentations of complications following mastectomy being loss in nipple or breast sensation, uneven breasts, loss in nipple or breast sensation, breast swelling, infection, and hematoma formation. Mastectomy breast cancer survivors who underwent breast reconstruction following their surgery reported a significantly higher rate of cosmetic satisfaction and overall choice satisfaction with surgery type in breast cancer survivors who underwent reconstruction versus those who did not. This was in line with current findings that breast reconstruction following mastectomy is associated with improved quality of life in breast cancer survivors [25]. However, our findings and prior studies suggest that there is a reported increase in operative and post-operative complications. These complications included marginal incision necrosis and capsular contracture, associated with mastectomy followed by immediate reconstruction compared to delayed reconstruction and mastectomy alone [25, 26]. Chronic pain findings were in line with prior research suggesting that reconstruction following mastectomy does not increase the incidence of chronic pain [27].

Aesthetic outcome following breast cancer surgery is an important component of breast cancer treatment and affects the quality of life through physical, social, emotional, and cognitive functioning [28]. Cosmetic results are affected by a wide range of factors including post-radiation skin changes or tissue fibrosis as well as post-surgical scar satisfaction, infection, or lymphedema presence, and reconstruction outcomes. Lymphedema was reported by both mastectomy and lumpectomy breast cancer survivors in our study. Axillary lymph nodes dissection and radiation are the most significant causes of breast swelling and post-surgical lymphedema [29]. Studies have shown that most lumpectomy breast cancer survivors are satisfied or very satisfied with their post-surgical aesthetic results with lower satisfaction associated with larger excised breast tissue and axillary lymph node removal [30]. Our research aimed to compare the post-surgical scar aesthetics satisfaction between mastectomy and lumpectomy breast cancer survivors and found similar findings to prior studies indicating that the satisfaction rate was significantly lower in those who underwent a mastectomy. There was, however, no significant difference in the overall surgical choice satisfaction rate between mastectomy and lumpectomy among breast cancer survivors with unrestricted choice of surgery.

This study was conducted by distributing the research questionnaire on several online breast cancer support groups. A limitation based on the bias of selecting only breast cancer survivors who participate in online support groups is possible. The survey platform was open to all participants without having a limitation to the number of times it can be accessed. Hence, the possibility of multiple submissions was not eliminated.

## Conclusions

When comparing mastectomy and lumpectomy, there were significant differences among cosmetic outcome and chronic pain prevalence. Patients that received lumpectomy were significantly more satisfied with their cosmetic outcomes when considering post-surgical scar but did report skin thickening as a common radiation side effect. Compared to lumpectomy, mastectomy is associated with lower rates of chronic pain including pain at the six months threshold and pain that is persistent and present up to the time of survey.

Although mastectomy patients had higher rates of reconstruction compared to lumpectomy patients, there was no significant difference in incidence of complications between breast reconstruction and non-reconstruction following surgery. Finally, overall patient surgical choice satisfaction showed no significant difference between mastectomy and lumpectomy patients. The composite findings will provide information that will aid future breast cancer patients in making a decision between having a mastectomy or a lumpectomy.
